# Preferences of healthcare workers for security personnel to prevent occupational violence: A discrete choice experiment

**DOI:** 10.1177/10519815251330539

**Published:** 2025-04-17

**Authors:** Sameera Senanayake, Jed Duff, Lita Jeffries, Joanna Griffiths, Ruvini Hettiarachchi, Pakhi Sharma, Sanjeewa Kularatna

**Affiliations:** 1 Duke-NUS Medical School, Health Services and Systems Research, Singapore; 2 Australian Centre for Health Services Innovation, School of Public Health and Social Work, Faculty of Health, Queensland University of Technology, Brisbane, QLD, Australia; 3 Centre for Healthcare Transformation, School of Public Health and Social Work, Faculty of Health, Queensland University of Technology, Brisbane, QLD, Australia; 4 Queensland Occupational Violence Strategy Unit, Brisbane, QLD, Australia; 5 Centre for the Business and Economics of Health, The University of Queensland, Brisbane, QLD, Australia

**Keywords:** occupational exposure, health personnel, workplace violence, security measures, choice behaviour, violence prevention and control

## Abstract

**Background:**

Occupational violence against healthcare workers is increasing worldwide. The role of security personnel in healthcare settings is critical, yet little is known about the attributes of security personnel that are most important to healthcare workers.

**Objective:**

This study seeks to identify the preferred attributes of security personnel from the healthcare professional's perspective.

**Methods:**

An online survey was administered to a representative sample of healthcare staff including health service managers, clinicians, and nurses at Metro North Hospital and Health Service in Queensland, Australia. We employed a convenience sampling method where the survey link was emailed to 780 healthcare staff members, and 179 participants responded. Using the Discrete Choice Experiment methodology, this study quantified individuals’ preferences. It explored several attributes of security personnel, including skills, uniform presence, coverage location, availability, level of professional development, and whether the security personnel are integrated within the clinical team.

**Results:**

Healthcare workers showed a strong preference for security personnel possessing interpersonal skills. They favoured personnel located within specific wards or units, available round-the-clock (24/7), and being embedded within the clinical team. Further, they strongly preferred personnel who have undergone extended training in professional development. Interestingly, the presence or absence of a security uniform did not have an impact on their preferences.

**Conclusion:**

The results of this study offer insights into the optimal attributes of security personnel from a healthcare workers’ perspective. Future violence prevention strategies can be designed taking into consideration these preferred attributes of security personnel, thus increasing the likelihood of their acceptance and success among healthcare professionals.

## Background

Occupational violence, a significant problem in healthcare settings, refers to an incident where a healthcare worker is abused or assaulted in their workplace.^1^ As per the World Health Organization, up to 38% of health workers suffer physical violence at some point in their careers, and some are threatened or exposed to verbal aggression.^2^ In the United States, a Violence Surveillance Study in 2012 found that more than half of the nurses surveyed reported having been exposed to either verbal or physical abuse within the preceding week.^3^ Another study conducted in Australia in 2017 found that, in the preceding six months, the emergency department staff had experienced verbal (88%) or physical (43%) assault.^4^ Occupational violence not only has a negative impact on the health and wellbeing of healthcare workers, but it may also affect patient care and safety.^5,6^ Therefore, it is critical for healthcare organisations to prioritise the prevention of occupational violence and provide resources and support for healthcare workers who experience it.

A proactive approach that addresses root causes and promotes a safe work environment is needed to prevent occupational violence in healthcare settings. Approaches including education, training, and increasing staff levels for example security personnel, are effective in reducing risks.^7,8^ Security personnel play a key role in preventing occupational violence by creating a safe and secure environment for healthcare workers, patients, and visitors.^9,10^ Several programmes have been implemented dedicated to recruiting and training security personnel at healthcare settings, such as the SafeCare programme in Canada provides training on communication, de-escalation techniques, situational understanding, and self-defence.^11^ In the United States, the International Association for Healthcare Security and Safety offers several training and certification programmes for healthcare security personnel.^12^ These programmes have stressed the importance of appointing security personnel in healthcare settings.

Occupational violence prevention has been identified as a priority area for Queensland Health, with increased incidence in recent years.^13^ To address occupational violence, the Queensland Occupational Violence Strategy Unit (QOVSTU) was started in 2016, and is a state-wide entity focused on developing, trialling, and implementing strategies to mitigate occupational violence in Queensland Health settings. The QOVSU piloted the ‘Ambassador’ initiative, a non-traditional security personnel in healthcare settings, at the Royal Brisbane and Women's Hospital.^14^ The aim of the Ambassador programme was to increase staff, patient, and visitor safety, and reduce violence in acute care settings through proactive and positive engagements between clinicians, patients, visitors, and healthcare security. The Ambassador engages with patients and visitors to prevent and/or reduce aggressive/disruptive behaviour, by employing appropriate verbal strategies through respectful, empathetic, and supportive interactions and reduce the occupational violence directed toward the healthcare workforce.^14^

This study specifically focuses on healthcare workers’ preferences for security arrangements, recognising the distinct and frontline role of security personnel in these environments. Their immediate response capabilities in violent situations underscore the necessity of aligning their attributes and roles with the expectations and needs of the healthcare staff. Focusing on these preferences is key to effectively tailoring the recruitment, training, and integration of security personnel, ultimately contributing to a more responsive and conducive workplace for healthcare professionals. Thus, while our study concentrates on one aspect of the broader spectrum of violence prevention strategies, it addresses a critical and often underrepresented component, offering deeper insights into healthcare staff preferences and needs to effectively scale and implement future programmes.

Discrete choice experiments (DCEs) have gained popularity as a robust choice method in fields of healthcare as it considerably contributes to policy making, health resource allocation, and addressing gaps in healthcare services.^15–17^ In a typical DCE, individuals are presented with several hypothetical health scenarios (choice sets), each containing several alternatives with different attributes (characteristics) for the individuals to choose from.^16^ DCE is a stated preference technique that is useful in determining weighted preferences of attributes from various stakeholders, including healthcare staff in a decision-making situation.^18^ Regarding recruiting security personnel, there may be many attributes (for example training, education, and physical or mental strength) that may be important from the perspective of healthcare workers, and the weighted preferences for each of these can be captured by DCEs. Few studies have used the DCE methodology in this field; however, they are not about preferences for recruiting or training security personnel.^19^

In response to the growing issue of occupational violence in healthcare, this study broadly aims to understand healthcare workers’ preferences for security personnel arrangements. Furthermore, it focuses on evaluating the preference of the Ambassador initiative compared to traditional security roles. By identifying the preferred attributes of security personnel, the study seeks to determine which aspects are most preferred in enhancing safety and reducing violence in healthcare settings. The insights gained will be crucial for developing targeted recruitment and training strategies, ensuring they align with the unique needs of healthcare staff and foster a safer, more supportive work environment.

## Methods

### Ethical considerations

This study received ethics clearance, and electronic informed consent was obtained from all individual participants. On the first page of the online survey, participants were asked to indicate their consent by selecting either “Agree” to participate or “Not Agree” to decline participation. Only those who selected “Agree” were granted access to the survey. Participants were encouraged to review the participant information sheet in full before proceeding, to ensure they were aware of the benefits and potential risks of providing their data for research purposes.

### Development of attributes and levels

The design was a non-labelled DCE study where respondents were presented with two hypothetical scenarios, known as choice sets, each containing six attributes. The selection and finalisation of the DCE attributes and levels were based on review of the literature, qualitative interviews with health service providers, a quantitative structured prioritisation exercise and an expert panel discussion.^20^ The five included attributes were: (i) skills, (ii) uniform, (iii) coverage, (iv) security availability, (v) training, and (vi) embedded with the clinical team. The attributes and a description of their levels are shown in Table 1.

**Table 1.** Included attributes and their associated levels.

Attribute description	Levels
Skills	o Physical restraint skills o Interpersonal skills (empathy, compassion, understanding, communication skills) o Risk observation skills
Uniform	o Security uniform o No security uniform
Coverage	o Located within the wards/ unit/department o Assigned across multiple wards o Located across the facility (Entire Hospital)
Security availability	o 24/7 (all shifts) o After hours only (After 4 pm and weekends) o On call
Training	o Basic level professional development o Mid-level professional development (de-escalation, diffuse, direct + communication training) o Extended professional development (trauma-informed care, de-escalation, negotiation, medical terminology, and conditions)
Embedded to the clinical team	o Yes o No

The Ambassador security personnel are characterised by several key attributes: possessing strong interpersonal skills, not wearing traditional security uniforms, being strategically positioned within wards, units, or departments, receiving mid-level and extended professional development training, and being integrally embedded within clinical teams. These features distinguish them from conventional security staff and underscore their tailored approach to managing occupational violence in healthcare environments.

### Experimental design

**Figure 1.**
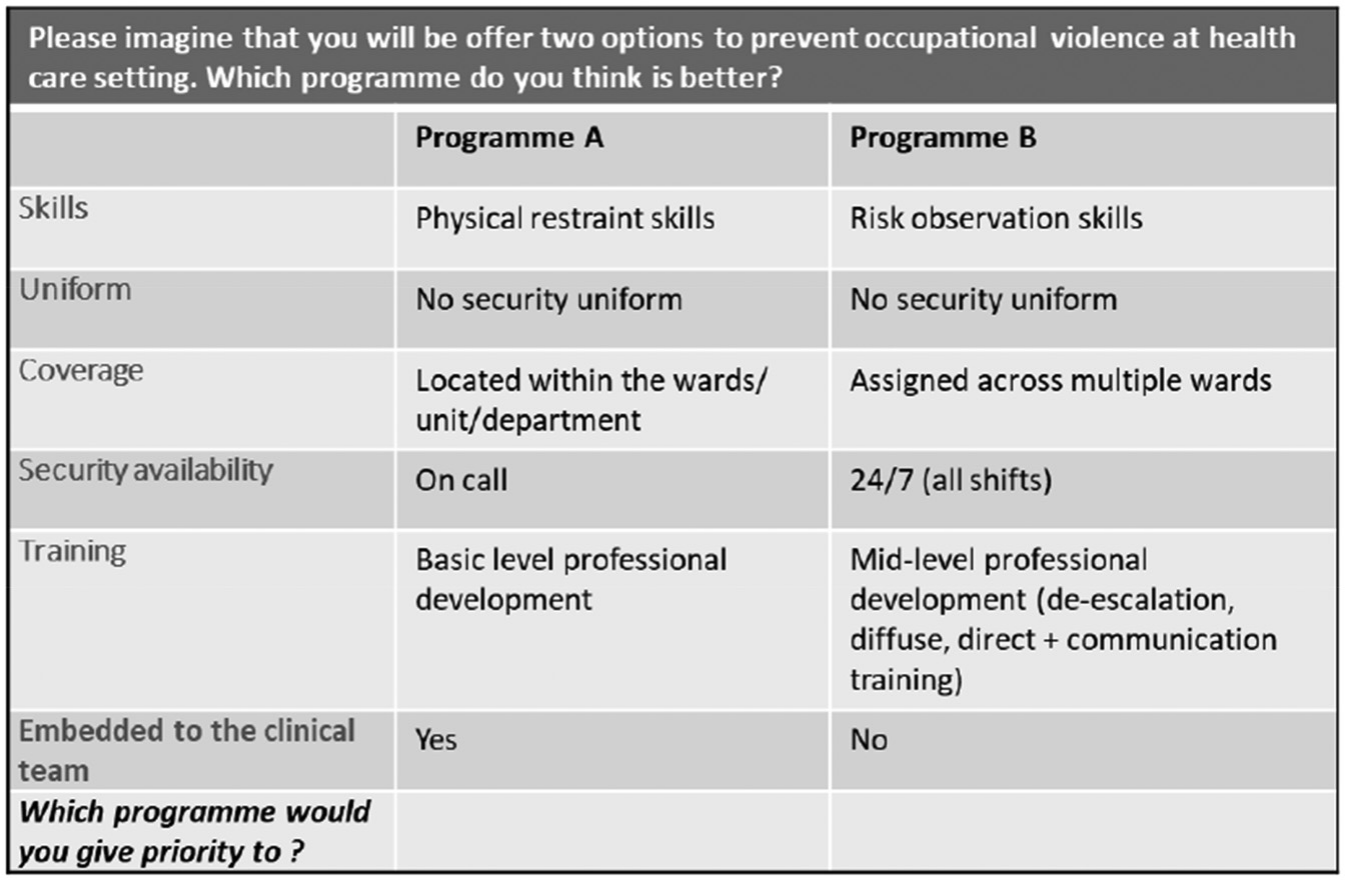
Example of a choice task seen by respondents.

The final attribute list had six attributes, with four attributes having three levels each and two attributes having two levels each. This would result in 324 (3^4 ^× 2^2^) possible profiles and 52,326 combinations of pairwise choice tasks [324(324-1)/2]. An example choice task is given in Figure 1. Since it is not feasible to present all possible combinations (n = 52,326) to all the respondents, 30 pairwise choice tasks were selected using Ngene software in a fractional factorial design. The 30 choice tasks make up the choice set. The main aim of using a fractional factorial design was to have a manageable number of choice tasks while maximising the design's statistical efficiency.^21^ Therefore, a multinomial logit model based on D-efficient fractional factorial design criteria (using the D-error value) was used to develop 30 pair-wise choice tasks using the design software Ngene. Evidence indicates that respondents can efficiently handle ten choice sets at a time.^22,23^ Therefore, the fractional factorial design was divided into three blocks so that a respondent would only answer ten from the 30 choice tasks in the fractional factorial design. Blocking is an accepted statistical technique in a DCE design that ensures an equal number of respondents per block.^24^ We used the modified Federov algorithm to develop the D-efficient design, which is known to develop designs with no dominant choice tasks.^25^

In the absence of prior information on the coefficients of the different attributes, small positive or negative priors or zero priors (non-informative priors) (Supplementary Table 1) were used to design the D-efficient design based on the following a priori hypotheses. We assumed that staff prefer security personnel:

- With interpersonal skills and risk observation skills.
- With no security uniform.
- Located within the wards/ unit/department.
- Available 24/7 (all shifts).
- Training in extended professional development (trauma-informed care, de-escalation, negotiation, medical terminology, and conditions).
- Embedded to the clinical team.

In addition to the D-error, attribute level overlap and attribute level balance were used to assess the DCE design. Attribute level overlap is when the same attribute level appears in both choice tasks, essentially eliminating this attribute from that choice task. Attribute level balance is the distribution of the attribute levels across the two choice tasks. Lower attribute level overlap and an equal distribution of levels indicate a better DCE design. Of the 30 choice tasks, none of the attributes had overlapping attribute levels (Supplementary Table 2) and the levels of the six attributes were almost equally distributed in both the choice tasks (Supplementary Table 2).

### Study population

The study population consisted of healthcare staff, including health service managers, clinicians, and nurses, at Metro North Hospital and Health Service in Queensland, Australia. Potential participants were contacted by the Queensland Occupational Violence Strategy Unit (QOVSU) team. An invitation email, including the participant information sheet and the survey tool, was sent to 780 healthcare staff members. Inclusion criteria were healthcare staff in the above categories employed within the Metro North Hospital and Health Service in Queensland. No formal exclusion criteria were applied in this study. All healthcare staff who were invited and consented to participate were included in the survey. Participants who did not provide consent were excluded automatically through the online platform. Those who wished to participate were directed to the consent form and survey through a survey link. Ultimately, responses were obtained from 179 participants.

This recruitment method was chosen primarily due to logistical and time constraints, which made more rigorous probability sampling methods impractical in this setting. Convenience sampling is commonly used in DCEs, particularly in time-sensitive environments like healthcare, where professionals often have limited availability. By leveraging the Queensland Occupational Violence Strategy Unit (QOVSU) team's established communication channels, we were able to efficiently reach a sample of healthcare staff. While these staff members are key stakeholders directly impacted by occupational violence and play critical roles in patient care and decision-making, we acknowledge that the participation rate was low (23%). While convenience sampling has limitations in generalisability, it ensured participation from individuals with direct experience and a vested interest in the study topic. This approach allowed us to balance feasibility within the operational constraints of the hospital environment while capturing meaningful insights into the preferences and needs of healthcare staff.

### Questionnaire design and data collection

The web based DCE survey had two parts. First, demographic data were collected (e.g., gender, age, education) to summarise the characteristics of the study participants. The second part contained the ten DCE tasks. In addition to these ten choice tasks per respondent, a repeated choice task and a dominant choice task were also included to assess the internal reliability and consistency of responses, creating 12 DCE choice tasks presented to each participant. A choice task with an apparent dominant option was presented at the beginning of the main DCE tasks. The proportion who got this dominant option correct was considered a proxy indicator of the internal reliability and consistency of responses. The third-choice task was repeated at the end of the ten main tasks. The proportion who got the same answer to the repeated tasks was also considered a proxy indicator of the internal reliability and consistency of responses.

The web based DCE survey was pre-tested aiming to ascertain comprehension and understanding of the online survey. We conducted a think-aloud exercise by administering the online survey to a sample of 10 healthcare staff members. These participants were selected using a convenient sampling method from the known contacts of the Queensland Occupational Violence Strategy Unit (QOVSU) team. This approach allowed us to gather immediate feedback from a group that was easily accessible and familiar with the context of the study. The participants were presented with one choice task at a time and requested to verbalise their thoughts in selecting the alternatives. They were requested to indicate if they faced difficulties in understanding the choice tasks or felt cognitively burdened by the numbers of attributes, levels, and choice tasks. Their feedback was used to refine the choice task scenario and wordings of few levels. Moreover, they noted that the survey was easy to understand. After addressing the changes, we proceeded to administering the DCE survey.

### Econometric model

A multinomial logit model under a random utility framework was used to analyse DCE data. The random utility framework assumes that the participants chose the alternative that maximised their utility. The utility function is estimated using the six attributes and a random error term. The coefficients in the MNL model indicates the relative importance of each independent variable (or attribute) on the dependent variable (preference of the individual). A negative coefficient indicates that the independent variable has a negative impact on the individual's preference, while a positive coefficient indicates a positive impact. This information can be used to determine which factors are most important in shaping the individual's preferences. The analysis was conducted in NLOGIT 5 software (https://www.limdep.com/products/nlogit). The validity of the responses was assessed using the percentage of respondents who got the repeat and dominant DCE tasks correct and by the average time taken to respond to the survey.

After testing several specifications for the discrete choice model, the below utility model was estimated:

$$\begin{array}{rl} V= & \beta_{0}+\beta_{1}SKILLS_{Interpersonal}+\beta_{2}SKILLS_{RiskObservation\;}\\ & \;+\beta_{3}NO\_UNIFORM+\beta_{4}COVERAGE_{WithinWards}\\ & \;+\beta_{5}COVERAGE_{AcrossWards}+\beta_{6}AVAILABILITY_{24/7}\\ & \;+\beta_{7}AVAILABILITY_{AfterHours}+\beta_{8}TRAINING_{Mid-level}\\ & \;+\beta_{9}TRAINING_{Extended}+\beta_{10}EMBEDDED\_IN\_CLINIC \end{array}$$

Where V is the observable relative utility and is a function of constant β_0_ and ten parameters measuring the effect of security personnel attributes described in Table 1.

## Results

### Demographic characteristics

A total of 179 participants responded to the survey (response rate 23%), and Table 2 describes the sample's demographic characteristics. There majority of the respondents were between 36 to 55 years (51%) and were females (76%). Nearly half the study participants (44%) were nurses.

**Table 2.** Sample characteristics.

Variable	Categories	Number (%)
Age in years	18–35 years	51 (28%)
	36–45 years	38 (21%)
	46–55 years	54 (30%)
	56–65 years	30 (17%)
	Prefer not to say	06 (3%)
Sex	Male	41 (23%)
	Female	137 (76%)
	Non-binary	1 (0.6%)
Staff category	Nurse	40 (22%)
	Specialist Nurse	36 (20%)
	Medical Officer	22 (12%)
	Nurse Unit Manager	3 (2%)
	Midwife	2 (1%)
	Other	76 (43%)

### Validity of the responses

The median time taken to respond to the survey was 8.5 min (inter-quartile range 7 to 11 min), which was within the expected time from the pilot study. The dominant and repeat tasks were correct in 85% and 90% of the responses, respectively.

### Discrete choice experiment results

Coefficients and the 95% confidence intervals of the MNL model is presented in Table 3. Compared to physical restraint skills, the respondents preferred the security personnel with interpersonal skills (coefficient 0.60 [95% CI: 0.31, 0.88]). Presence or absence of a security uniform did not have a significant impact on participants’ preferences. Results indicated that security personnel located within the wards/unit/department was preferred (coefficient 0.27 [95% CI: 0.01, 0.55]), compared to being located across the facility (Entire Hospital). Availability of the security personnel 24/7 had the largest coefficient (1.85 [95% CI: 1.58, 2.12]), indicating that it was the most preferred attribute of a security personnel. Training was also found be a preferred attribute, with mid-level professional development having a coefficient of 1.49 (95% CI: 1.19, 1.79) and extended professional development having a coefficient of 1.68 (95% CI: 1.37, 1.99). Being embedded within the clinical team was also found to be a preferred attribute (coefficient 0.28 [95% CI: 0.06, 0.51]).

**Table 3.** Multinomial logit model estimates.

	MNL model *Coefficient (95% CI)*
Constant	−2.3 [−3.0, −1.7]
Skills	
Physical restraint skills	Reference
Interpersonal skills	0.60 [0.31, 0.88]
Risk observation skills	−0.12 [−0.4, 0.16]
Uniform	
Security uniform	Reference
No security uniform	−0.04 [−0.26, 0.17]
Coverage	
Located within the wards/ unit/department	0.27 [0.01, 0.55]
Assigned across multiple wards	−0.07 [−0.35, 0.22]
Located across the facility (Entire Hospital)	Reference
Security availability	
24/7 (all shifts)	1.85 [1.58, 2.12]
After hours only (After 4 pm and weekends)	0.49 [0.21, 0.76]
On call	Reference
Training	
Basic level professional development	Reference
Mid-level professional development	1.49 [1.19, 1.79]
Extended professional development	1.68 [1.37, 1.99]
Embedded to the clinical team	
Yes	0.28 [0.06, 0.51]
No	Reference

## Discussion

This study aimed at eliciting healthcare workers’ preferences for an ideal security personnel in a healthcare setting. Healthcare workers preferred security personnel with interpersonal skills compared to physical restraint skills, located within the wards or units rather than across the entire facility, available 24/7, and embedded within the clinical team. They also preferred security personnel who have undergone extended professional development training compared to mid-level training. Presence or absence of a security uniform did not have a significant impact on preferences. Therefore, the results indicate that the respondents preferred the characteristics of the ‘Ambassador’ initiative compared to those of more traditional security personnel.

The use of DCE methodology is relatively new in fields of healthcare; however, it has proven its significance in health service improvements, resource allocation, and guiding policymakers.^15,17,18,26^ It uses the ‘random utility framework’ enabling individuals to choose from the best options available. DCEs offer precise insights into individual preferences for security personnel attributes, a key component in addressing occupational violence in healthcare. While DCEs provide quantifiable data on specific attributes, they differ from methods like focus groups or deliberative polling, which enable collective discussion and consensus. Qualitative methods, although insightful for capturing personal experiences, face challenges in validity and reliability, such as varying data interpretation and potential researcher influence.^27,28^ Our study's aim was to compare individual preferences for traditional security personnel and those under the Ambassador initiative, a focus not readily achievable through group consensus methods. The DCE approach, while distinct in its individualistic data collection, complements the broader understanding of community needs gained from qualitative research, making it a valuable tool in designing effective occupational violence prevention strategies in healthcare. Literature suggests that DCEs are a valuable tool for rational decision-making in healthcare settings, providing insights into preferences by requiring respondents to make explicit trade-offs​​. DCEs have been widely used in various healthcare settings, including workforce planning and policy making, underscoring their value in capturing healthcare professionals’ preferences.^29^

Previously, a DCE analysis has not been conducted explicitly on healthcare workers regarding their preferences for a security personnel; however, one DCE study states the preferences of nurses and midwives for their jobs and the results indicates that one of the most important attributes were processes to deal with violence and bullying. Moreover, nurses and midwives were willing to forgo 16% of their annual salary for the implementation of adequate processes to deal with violence and bullying.^19^ This indicates the importance of this issue and the reforms needed within health services.

This study assessed several key attributes that, according to healthcare workers, are essential for an ideal security personnel to have. In our study, healthcare workers preference was interpersonal skills over physical and risk observation skills. Few studies highlight the presence of insufficient interpersonal skills in security personnel and the need for organisations to train them with such soft skills.^30,31^ Being able to communicate with those who may cause violence may help in judging the situation beforehand and taking appropriate actions. In our study, presence or absence of a security uniform did not have a significant impact on preferences, which is contrary to the evidence in some literature. For instance, a study in Canada gauged views of hospital security staff and the interviews indicated that uniforms define a clear image, authority to the public, and that it can be essential to gain respect from public.^32^ Other studies examined the perception of healthcare attire of all staff and not specifically of security personnel.^33,34^

In our study, entire facility coverage was selected as the reference scenario, however staff indicated a preference for the security role to be located within specific wards or units. This highlights the challenge that healthcare facilities face in allocating and deploying security personnel.^35^ It may be implied that for future programmes, coverage should be prioritised as per the requirements of the facility and the number of staff available to fulfill it. In terms of availability, 24/7 readily available security is preferred as per our study. Providing 24/7 embedded security requires a significant resource allocation. A study in the US in emergency departments, a traditionally high-risk area, found that only 63% had 24-h security personnel availability.^36^ Other evidence recommends that the scheduling of security personnel should be based on a risk assessment using with objective criteria.^35^ However, this may also depend on the number of staff available and the priority at the time.

A survey administered to security personnel themselves indicated that the most common suggestion to prevent violence was adequate training.^37^ Prior security experience and training courses were important factors within training as well.^36^ A study conducted in Western Sydney Local Health District in New South Wales indicated that easily accessible and repeatable experiential training in managing Code Black (a threat to personnel) was vital.^38^ Interviews in the same study suggested that nearly one-third of staff were not formally trained and only had a vague understanding about initiating violence prevention and management interventions. The authors advocate that important aspects of training include proactively addressing potential behavioural challenges involving anticipating signs of agitation; employing early de-escalation techniques; modifying the environment to enhance safety, for instance, removing sharp objects, ensuring accessible exits and pathways, maintaining a safe distance from the patient, and keeping doors open.^38^

Findings from our study indicate that healthcare workers prefer security personnel to be embedded within the clinical team. This change would represent a variation to the traditional model used by many healthcare security services. Commonly, security services will operate independently from the clinical wards and units in both their role, functions, and governance.^39^ An embedded model has both advantages and disadvantages associated to it. An advantage, for example, may be the effectiveness of the programme by actively involving the clinical team in violence management and prevention.^39^ An identified disadvantage of the model is the potential for fatigue and burnout among security personnel embedded in high risk areas with frequent incidents of violence.^39^

Our findings suggest that the recruitment of security personnel possessing essential attributes could play a significant role in mitigating occupational violence and fostering a safe and secure environment for healthcare workers. Giving due consideration to the needs of healthcare workers who actively contribute to establishing hospitals and other healthcare facilities as healing environments is important. Consequently, healthcare organisations should prioritise addressing the escalating issue of occupational violence experienced by their workforce. Future initiatives aimed at preventing violence among staff should incorporate comprehensive measures such as enhanced training programmes and skill development courses and ensuring sufficient coverage and availability of security personnel. Additionally, embedding security personnel within the clinical team may prove to be a suitable approach. To accurately determine the requirements of healthcare staff in terms of their workplace safety, it would be beneficial to conduct further preference studies utilising appropriate choice methods within healthcare organisations. These studies can help in accurately predicting the specific needs and preferences of staff members to create a secure work environment.

### Strengths and limitations of this study

This study employed an extensive attribute development procedure including literature review, qualitative in-depth interviews with health service providers, a quantitative structured prioritisation exercise and an expert panel discussion.^20^ Conducting an appropriate attribute development procedure that includes a sound qualitative analysis improves transparency and validity for producing subsequent quantitative evidence for DCEs.^40^

There were few limitations in this study. The findings are limited to Queensland, Australia as the data was collected from healthcare staff at the Metro North Hospital and Health Service (HHS), Queensland. As a result, the findings may not be generalisable to other regions or healthcare settings. However, given the lack of existing evidence in this area, these results offer valuable initial insights that can inform future research across diverse geographic locations. The priors were not generated via pilot study, due to the absence of prior information on the coefficients of the different attributes. Instead, small positive or negative priors or zero priors (non-informative priors) were used. Another limitation of this study is the relatively low response rate of 23%, which is consistent with typical response rates among healthcare workers due to the demanding nature of their roles and time constraints. While this may limit the generalisability of the findings, convenience sampling and low response rates are common in DCEs within healthcare settings. Despite this, the study still provides valuable insights into the preferences of healthcare staff. We have acknowledged this limitation transparently in the discussion to allow readers to assess the relevance of the findings to their own contexts without overstating the results. Future studies should consider strategies to improve participation, such as offering incentives or sending follow-up reminders to increase response rates.

## Conclusion

Incorporating the most relevant attributes and needs of healthcare workers into policies and protocol development ensures that decision-making processes are evidence-based and aligned with staff expectations. For instance, prioritising interpersonal skills in security personnel recruitment and designing comprehensive training programmes that include trauma-informed care and de-escalation techniques can enhance the effectiveness of violence prevention strategies. Additionally, embedding security personnel within clinical teams and ensuring their availability round-the-clock could foster a safer work environment and improve staff confidence in security measures. Furthermore, healthcare organisations should actively involve healthcare workers in the development and refinement of security protocols to ensure that these measures address their practical concerns and operational challenges. Regular feedback mechanisms and continuous monitoring of implemented measures will ensure that the policies remain adaptive to evolving workplace dynamics and challenges. By integrating these attributes into a cohesive framework, organisations can create tailored, effective violence prevention strategies that contribute to a safer and more supportive healthcare environment.

## Supplemental Material

sj-docx-1-wor-10.1177_10519815251330539 - Supplemental material for Preferences of healthcare workers for security personnel to prevent occupational violence: A discrete choice experiment Supplemental material, sj-docx-1-wor-10.1177_10519815251330539 for Preferences of healthcare workers for security personnel to prevent occupational violence: A discrete choice experiment by Sameera Senanayake, Jed Duff, Lita Jeffries, Joanna Griffiths, Ruvini Hettiarachchi, Pakhi Sharma and Sanjeewa Kularatna in WORK
