# Supplementary material for: Preferences of healthcare workers for security personnel to prevent occupational violence: A discrete choice experiment
Source: Work. 2025 Apr 17;81(4):3285–93. doi: 10.1177/10519815251330539 (PMC12287554; doi:10.1177/10519815251330539)
Supplement: sj-docx-1-wor-10.1177_10519815251330539 - Supplemental material for Preferences of healthcare workers for security personnel to prevent occupational violence: A discrete choice experiment [file sj-docx-1-wor-10.1177_10519815251330539.docx]

**Supplementary table 1:** Estimates used to design the choice tasks for the final survey

| **Attribute** | **Priors used in the Ngene design** |
| --- | --- |
| Skills |  |
| Physical restraint skills | Reference |
| Interpersonal skills | 0.00001 |
| Risk observation skills | 0.00002 |
| Uniform |  |
| Security uniform | Reference |
| No security uniform | 0.00001 |
| Coverage |  |
| Located within the wards/ unit/department | 0.00002 |
| Assigned across multiple wards | 0.00001 |
| Located across the facility (Entire Hospital) | Reference |
| Security availability |  |
| 24/7 (all shifts) | 0.00002 |
| After hours only (After 4 pm and weekends) | 0.00001 |
| On call | Reference |
| Training |  |
| Basic level professional development | Reference |
| Mid-level professional development (de-escalation, diffuse, direct + communication training) | 0.00001 |
| Extended professional development (trauma-informed care, de-escalation, negotiation, medical terminology, and conditions) | 0.00002 |
| Embedded to the clinical team |  |
| Yes | 0.00001 |
| No | Reference |
|  |  |
| *Measures of efficiency* |  |
| D-error | 0.201 |

**Supplementary table 2:** Description of the final D-efficient design

|  | **DCE design used for the final survey** | |
| --- | --- | --- |
| Number of rows | 30 | |
| Blocks | 3 | |
|  |  | |
| *Attribute overlap* |  | |
| Skills | 0/30 | |
| Uniform | 0/30 | |
| Coverage | 0/30 | |
| Security availability | 0/30 | |
| Training | 0/30 | |
|  |  | |
| ***Attribute level balance*** | **DCE design used for the final survey** | |
|  | ***Choice task 1*** | ***Choice task 2*** |
| Skills |  |  |
| Physical restraint skills | 9/30 | 9/30 |
| Interpersonal skills | 9/30 | 12/30 |
| Risk observation skills | 12/30 | 9/30 |
| Uniform |  |  |
| Security uniform | 15/30 | 15/30 |
| No security uniform | 15/30 | 15/30 |
| Coverage |  |  |
| Located within the wards/ unit/department | 9/30 | 12/30 |
| Assigned across multiple wards | 12/30 | 9/30 |
| Located across the facility (Entire Hospital) | 9/30 | 9/30 |
| Security availability |  |  |
| 24/7 (all shifts) | 9/30 | 12/30 |
| After hours only (After 4 pm and weekends) | 12/30 | 9/30 |
| On call | 9/30 | 9/30 |
| Training |  |  |
| Basic level professional development | 9/30 | 9/30 |
| Mid-level professional development | 12/30 | 12/30 |
| Extended professional development | 9/30 | 9/30 |
| Embedded to the clinical team |  |  |
| Yes | 18/30 | 18/30 |
| No | 12/30 | 12/30 |
